# Dysregulation of ribosome-related genes in ankylosing spondylitis: a systems biology approach and experimental method

**DOI:** 10.1186/s12891-021-04662-2

**Published:** 2021-09-14

**Authors:** Arezou Lari, Hamid Gholami Pourbadie, Ali Sharifi-Zarchi, Maryam Akhtari, Leila Nejatbakhsh Samimi, Ahmadreza Jamshidi, Mahdi Mahmoudi

**Affiliations:** 1 Systems Biomedicine Unit, Pasteur Institute of Iran, Tehran, Iran; 2 Rheumatology Research Center, Shariati Hospital, Tehran University of Medical Sciences, PO-BOX: 1411713137, Kargar Ave, Tehran, Iran; 3 Department of Physiology and Pharmacology, Pasteur Institute of Iran, Tehran, Iran; 4 Department of Computer Engineering, Sharif University of Technology, Tehran, Iran; 5 Inflammation Research Center, Tehran University of Medical Sciences, Tehran, Iran

**Keywords:** Ankylosing spondylitis, Ribosomes, Microarray analysis, RNA-seq

## Abstract

**Background:**

Ankylosing spondylitis (AS) is an autoimmune rheumatic disease. Few candidate gene associations have been reported for AS and the current understanding of its pathogenesis remains still poor. Thus, the exact mechanism of AS is needed to urgently be disclosed. The purpose of this study was to identify candidate genes involving in AS disease.

**Methods and results:**

GSE25101 publicly available microarray and GSE117769 RNA-seq datasets of AS patients were obtained for bioinformatics analyses. Gene set enrichment analysis showed that in the microarray dataset, the ribosome pathway was significantly up-regulated in AS compared with controls. Furthermore, some ribosomal components demonstrated overexpression in patients in the RNA-seq dataset. To confirm the findings, 20 AS patients and 20 matching controls were selected from the Rheumatology Research Center clinic, Shariati Hospital. PBMCs were separated from whole blood and RNA contents were extracted. Following the results of datasets analysis, the expression level of *rRNA5.8S* pseudogene, *rRNA18S* pseudogene, *RPL23*, *RPL7*, and *RPL17* genes were measured through real-time PCR. Our findings showed dysregulation of *rRNA5.8S* and *rRNA18S* pseudogenes, and also the *RPL17* gene in patients.

**Conclusion:**

Considering that genes involved in ribosome biogenesis contributed to some AS-associated biological processes as well as diseases that have comorbidities with AS, our results might advance our understanding of the pathological mechanisms of ankylosing spondylitis.

**Supplementary Information:**

The online version contains supplementary material available at 10.1186/s12891-021-04662-2.

## Introduction

Ankylosing spondylitis (AS) is the major subtype of spondyloarthropathies, which is one of chronic inflammatory arthritis [1, 2]. The characteristic symptoms of AS particularly are back pain, spinal stiffness, and loss of spinal mobility [3, 4]. Human leukocyte antigen (HLA) B27 is one of the most convincing genetic biomarkers associated with AS. However genetic association studies reveal that HLA-B27 only contributes to approximately 20% of AS heritability and 30% of the overall risk for AS [5, 6]. Previous studies suggested that AS is a multifactorial disease and the susceptibility to this disorder may be own to genetic and environmental factors [7]. Nevertheless, few genes associated with the disease were identified and the actual cause of AS has remained unclear [8]. Therefore, there is a crucial necessity to investigate the molecular mechanisms of AS in order to find more relevant biomarkers (s). Recent studies investigated other genes and inflammatory biomarkers related to the AS pathogenesis. Environmental factors through altering epigenetics mechanisms alongside with genetics polymorphisms control different gene expressions. Hence, altered gene expression profiles that differentiate disease from the healthy condition can be used as a basis of immuno-pathological mechanisms involved in the pathogenesis of AS [9, 10]. High-throughput genomics technologies such as microarrays and RNA sequencing allow analyzing gene expression profiles of thousand genes and provide deep insight into the interaction and network of the genes during complex diseases [11].

Eukaryotic ribosomes are the organelles consist of four ribosomal RNAs (rRNAs) and around 80 ribosomal proteins (RPs), whose function is to organize spatiotemporal control of gene expression. The RPs organize in small (40S) and large (60S) subunits and mostly are called with ‘S’ and ‘L’ letters, respectively [12, 13]. Ribonucleoprotein (RNP) components such as ribosomes are often targeted in various autoimmune disorders and recognized by B cells derived auto-antibodies [14, 15]. It was also demonstrated that perturbed expression of the RPs and rRNAs occurs in numerous human disorders, notably, in many cancers, ribosomopathies, and autoimmune diseases [16, 17].

In the current study, by expression profiling of AS-transcriptomes datasets and experimental analysis, we confirmed the dysregulation of some ribosomal genes in the disease. Ribosomes have traditionally been viewed as invariant [12], and their relationship to AS is not yet known, therefore, the finding of different expressions of ribosomal components in AS patients compared to the control group was interesting.

## Methods and donors

### Gene expression analysis

#### Microarray data analysis

The expression profile of GSE25101 which included 16 AS and 16 healthy samples was obtained from Gene Expression Omnibus (GEO) database. Characteristics of subjects involved in the microarray dataset are represented in Supplementary file 1 [18]. Following quantile normalization of microarray dataset, the limma package was used to determine differentially expressed genes between the AS and control groups with empirical Bayes analysis [19, 20]. Gene set enrichment analysis was conducted on normalized and log2 transformed data with the Gene Set Enrichment Analysis (GSEA) tool using curated genes from KEGG dataset (category C2) of MSigDB database [21].

#### RNA sequencing data analysis

For ribosomal RNA expression analysis, we used a publicly available dataset consisting of 120 samples with four phenotypes, namely; Rheumatoid arthritis, Ankylosing spondylitis, Psoriatic arthritis, and Healthy (GEO Accession: GSE117769 [22]). We analyzed all 8 ankylosing spondylitis samples in the dataset, along with an equal number of sex and age-matched control samples (Samples characteristics of RNA-seq dataset are shown in Table 1 and detailed information of patient samples are represented in Supplementary file 2). The RAW data of all selected samples were downloaded from EMBL-EBI (The European Bioinformatics Institute, PRJNA483133 accession number). The obtained RNA-seq libraries were processed by Trimmomatic version 0.38 to removed adapters, low-quality bases and read smaller than 50 bp (ILLUMINACLIP: LEADING:3 TRAILING:3 TruSeq3-SE:2:30:10 SLIDINGWINDOW:4:15 MINLEN:50), and the quality of all libraries was checked with FastQC, before and after trimming [23]. Then, the Salmon index file was built based on Ensembl (version 95) reference transcriptome annotation using k-mers of length 31, and the trimmed reads quantified using quasi-mappings of Salmon tool [24, 25]. We used tximport (version 1.10.1) to convert transcript-level to gene-level count estimates [26]. All differential expression analyses were performed with DESeq2 1.22.2 based on the negative binomial distribution, considering only the transcripts which had a TPM (transcripts per million) ≥1 in all samples [27].

**Table 1:** Characteristics of patients and healthy samples elected from the RNA-seq dataset

AS				Health			
Samples	Gender	Age	Download_Link	Samples	Gender	Age	Download_Link
GSM3308485	Female	28	*ftp://ftp.sra.ebi.ac.uk/vol1/fastq/SRR761/002/SRR7610232/SRR7610232.fastq.gz*	GSM3308520	Female	29	*ftp://ftp.sra.ebi.ac.uk/vol1/fastq/SRR761/007/SRR7610267/SRR7610267.fastq.gz*
GSM3308486	Female	47	*ftp://ftp.sra.ebi.ac.uk/vol1/fastq/SRR761/003/SRR7610233/SRR7610233.fastq.gz*	GSM3308447	Female	47	*ftp://ftp.sra.ebi.ac.uk/vol1/fastq/SRR761/004/SRR7610194/SRR7610194.fastq.gz*
GSM3308475	Female	33	*ftp://ftp.sra.ebi.ac.uk/vol1/fastq/SRR761/002/SRR7610222/SRR7610222.fastq.gz*	GSM3308522	Female	32	*ftp://ftp.sra.ebi.ac.uk/vol1/fastq/SRR761/009/SRR7610269/SRR7610269.fastq.gz*
GSM3308483	Female	41	*ftp://ftp.sra.ebi.ac.uk/vol1/fastq/SRR761/000/SRR7610230/SRR7610230.fastq.gz*	GSM3308518	Female	41	*ftp://ftp.sra.ebi.ac.uk/vol1/fastq/SRR761/005/SRR7610265/SRR7610265.fastq.gz*
GSM3308488	Male	45	*ftp://ftp.sra.ebi.ac.uk/vol1/fastq/SRR761/005/SRR7610235/SRR7610235.fastq.gz*	GSM3308530	Male	57	*ftp://ftp.sra.ebi.ac.uk/vol1/fastq/SRR761/007/SRR7610277/SRR7610277.fastq.gz*
GSM3308489	Male	35	*ftp://ftp.sra.ebi.ac.uk/vol1/fastq/SRR761/006/SRR7610236/SRR7610236.fastq.gz*	GSM3308519	Male	38	*ftp://ftp.sra.ebi.ac.uk/vol1/fastq/SRR761/006/SRR7610266/SRR7610266.fastq.gz*
GSM3308515	Male	32	*ftp://ftp.sra.ebi.ac.uk/vol1/fastq/SRR761/002/SRR7610262/SRR7610262.fastq.gz*	GSM3308537	Male	31	*ftp://ftp.sra.ebi.ac.uk/vol1/fastq/SRR761/004/SRR7610284/SRR7610284.fastq.gz*
GSM3308516	Male	48	*ftp://ftp.sra.ebi.ac.uk/vol1/fastq/SRR761/003/SRR7610263/SRR7610263.fastq.gz*	GSM3308541	Male	59	*ftp://ftp.sra.ebi.ac.uk/vol1/fastq/SRR761/008/SRR7610288/SRR7610288.fastq.gz*

### Experimental section

#### Sample selection

In this study, 20 AS patients were selected from the outpatient rheumatology Research Center (RRC) clinic, Shariati Hospital, Tehran, Iran. All diagnoses of AS by experienced Rheumatologists were made based on the modified New York criteria [28]. The patient group includes 15 men and 5 women with an average age of 35 years. The average duration since diagnosis is 8.7 years (more demographic characteristics are represented in Table 2). The patients were assessed for functional capacities and disease severity by a protocol based on the Assessment of Spondyloarthritis International Society (ASAS) core set [29]; Disease activity was evaluated by Bath Ankylosing Spondylitis Disease Activity Index (BASDAI) and only active AS patients with BASDAI ≥4 were selected [30]; damage or deformity of the spine was assessed by Bath Ankylosing Spondylitis Metrology Index (BASMI) [31]; Disease function was measured by Bath Ankylosing Spondylitis Functional Index (BASFI) [32]. No biologic-treated patients were included. For the control group, 20 healthy persons who matched in age and gender with the patients were selected and they should have no clinical evidence of any type of autoimmune disorders in themselves and their family. Signed informed consent was obtained from all the participants. The study procedure is confirmed by the Ethics Committee of Tehran University of Medical Sciences.

**Table 2:** Baseline characteristics of AS patients and healthy controls

Property	AS patients (***n*** = 20)	Healthy controls (***n =*** 20)
Male/ Female	15/5 (75%/25%)	15/5 (75%/25%)
Age	35 ± 11.3	36 ± 7.4
BASDAI	6.4 ± 2	–
BASFI	4.5 ± 2.6	–
BASMI	3.2 ± 1.2	–
Disease duration	8.7 ± 9.8	–
HLA-B27 positive	80%	0
ESR	29.3 ± 25.2	–
ASQol	9.6 ± 4.8	–

*AS* ankylosing spondylitis, *BASDAI* Bath Ankylosing Spondylitis Disease Activity Index, *BASFI* Bath Ankylosing Spondylitis Functional Index, *BASMI* Bath Ankylosing Spondylitis Metrology Index, *HLA-B27* Human Leukocyte Antigen (subtypes B*2701–2759), *ESR* Erythrocyte Sedimentation Rate, *ASQol* Ankylosing Spondylitis Quality of Life

#### Sample preparation and quantitative real time PCR

Five-milliliter blood was collected from all subjects in test tubes containing ethylenediamine tetra-acetic acid (EDTA). Samples were transferred to the laboratory on ice and possessed in about 2–3 h from the collection. PBMCs were separated from the whole blood by utilizing Ficoll-Hypaque gradient (Innotrain, Germany). RNA extraction was performed using the High Pure RNA Isolation Kit (Progen lab, Germany). Before cDNA synthesis, the concentration of extracted RNA was quantified by spectrophotometry (NanoDrop, Thermo Fisher Scientific, USA). According to Roche cDNA Synthesis Kit’s protocols, isolated RNA was converted to cDNA. Gene expression was measured by using the SYBR Green method for quantitative Real-time polymerase chain reaction (PCR) (Applied Biosystems, Foster City, USA). The specific primer sequences were shown in Table 3. Comparative Ct method was used for the relative gene expression analysis and Hypoxanthine phosphoribosyl transferase 1 (HPRT1) was used as a housekeeping gene for normalization. The expression data did not pass the normality test, so the Mann–Whitney test was used for mRNA expression analysis. The result with *p-value* of lower than 0.05 was considered significant.

**Table 3:** Sequence of primers used in qPCR in PBMC

Primer name	Forward primer	Reverse primer	Product size (bp)
***RPL23***	TCCGGATTTCCTTGGGTCTTC	CGTCCCTTGATCCCCTTCAC	103
***RPL17***	TGAAACTGCTCAGGCCATCAA	GTGTCCAGCCCCATTGCTTG	149
***RPL7***	GCCCTTCAAATTGTCTTCTCCAC	AATAAGCCTGTTGATCTGGTCCTC	106
***rRNA18S pseudogene***	TGGTGGAGTGATTTGTCTGGT	CATCTAAGGGCATCACAGACG	107
***rRNA5-8S pseudogene***	GGCTCCTGCGTTGATGAAGA	GCAAGTGCGTTCGAAGTGT	90
***HPRT1***	GGTGAAAAGGACCCCACGAA	AGTCAAGGGCATATCCTACAACA	92

*PBMC* Peripheral blood mononuclear cell, *RPL* Ribosomal protein, *HPRT1* Hypoxanthine Phosphoribosyltransferase 1

## Results

### Bioinformatics analysis outcomes

**Fig. 1:**
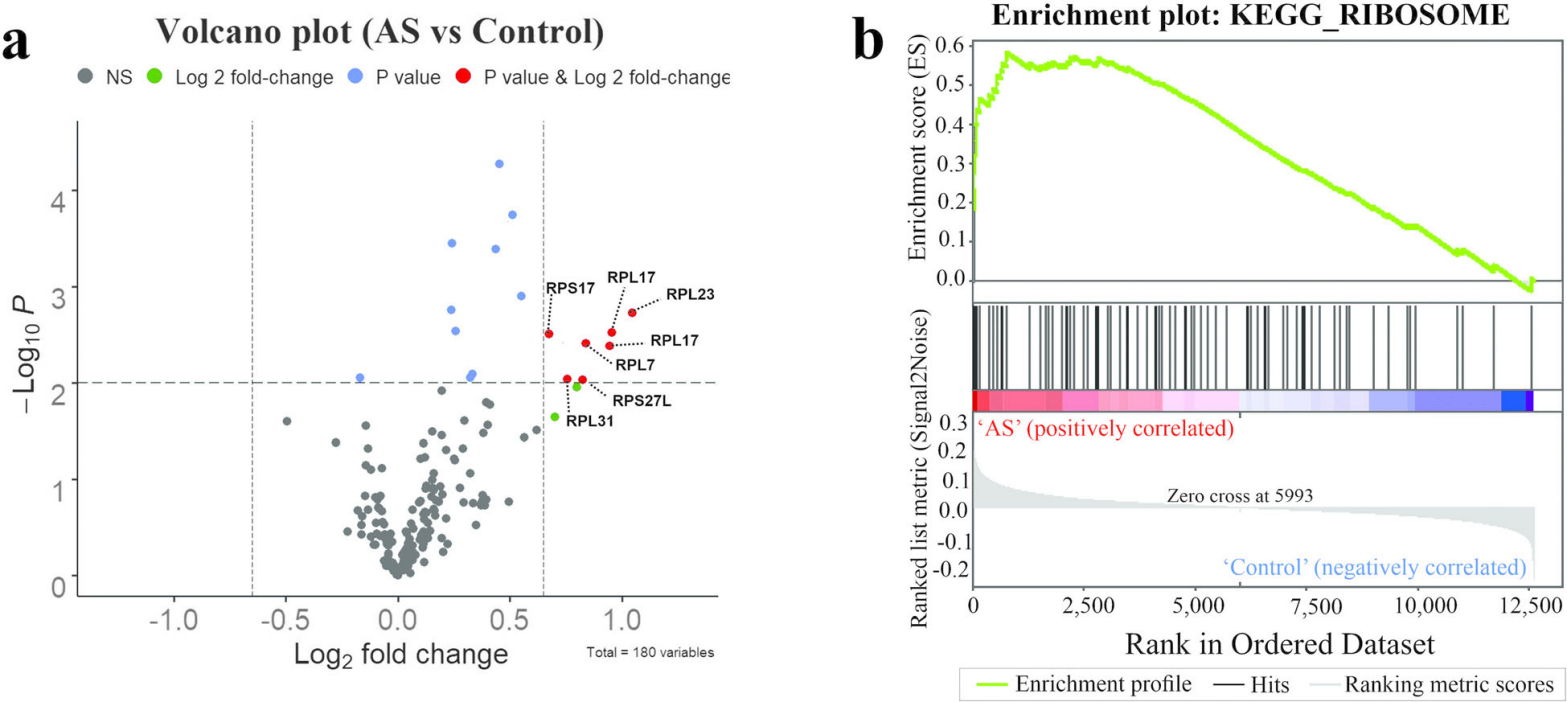
(a) In the Volcano plot, the x-axis indicates fold change and the y axis indicates minus the log of *p*-value. Significant expression levels using the 10–3 *p-value* and 0.65-fold change cutoff are indicated respectively by “blue” and “green”, interest genes that passed both thresholds are indicated by “red”, and no significant difference is indicated by “black”. (b) Enrichment plots from Gene Set Enrichment Analysis (GSEA). The plot demonstrated the ribosome pathway was enriched significantly in the microarray dataset

The analysis of the microarray dataset identified that ribosome pathway was significantly enriched by utilizing the GSEA method (normalized enrichment score (NES) = 2.18, false discovery rate (FDR *p-value*) = 0.000, and *p-value* = 0.000) (Fig. 1b). Figure 1a is a volcano plot of all genes (represented by dots) involving in the ribosomal pathway. The interest genes which is statistically significant as evidenced by the *p-values* and the fold change threshold in the microarray dataset are denoted by hexagon dots. The plot demonstrates higher expression profile changes of the ribosome pathway’s genes in the AS group. Three genes with the highest differentially expression included *RPL23* (Log2Fc = 1.04, *p-value* = 0.001), *RPL17* (Log2Fc = 0.9, *p-value* = 0.003), and *RPL7* (Log2Fc = 0.8, *p-value* = 0.003) were selected for the experimental section.

**Fig. 2:**
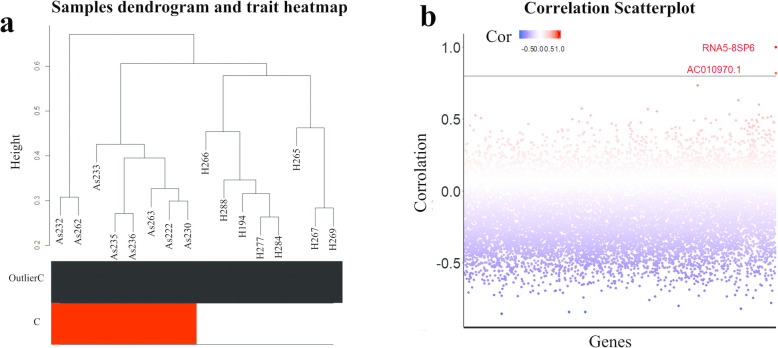
(a) Samples dendrogram and trait heatmap: Dendrogram for hierarchical cluster analysis of AS and healthy samples. Based on the trait heatmap there was no outlier. White represents H (Healthy) and red represents AS (Ankylosing spondylitis). (b) Correlation scatterplot: Correlation of rRNA5.8SP6 (as the most significant gene) with all other genes in the RNAseq dataset was measured by the Pearson method

RNA-seq analysis with DESeq2 confirmed that all of the significant genes (more than a thousand genes with *p-value* < 0.01) in the dataset can distinct very clearly between two groups (Fig. 2a; samples dendrogram and trait heatmap). In line with the notable expression trend of ribosomal genes in the microarray dataset, it found that ENST00000515896.1 which is a pseudogene of rRNA5.8S is one of the most significant genes in the RNA-seq dataset with Log2FC = 3.67 and *p-value* = 0.0001. Interestingly the gene has the highest correlation with another gene called AC010970.1(Log2Fc = 2.57, *p-value* = 0.001) which is an 18S ribosomal RNA pseudogene (Fig. 2b).

### Evaluation of mRNA expressions of selected genes

**Fig. 3:**
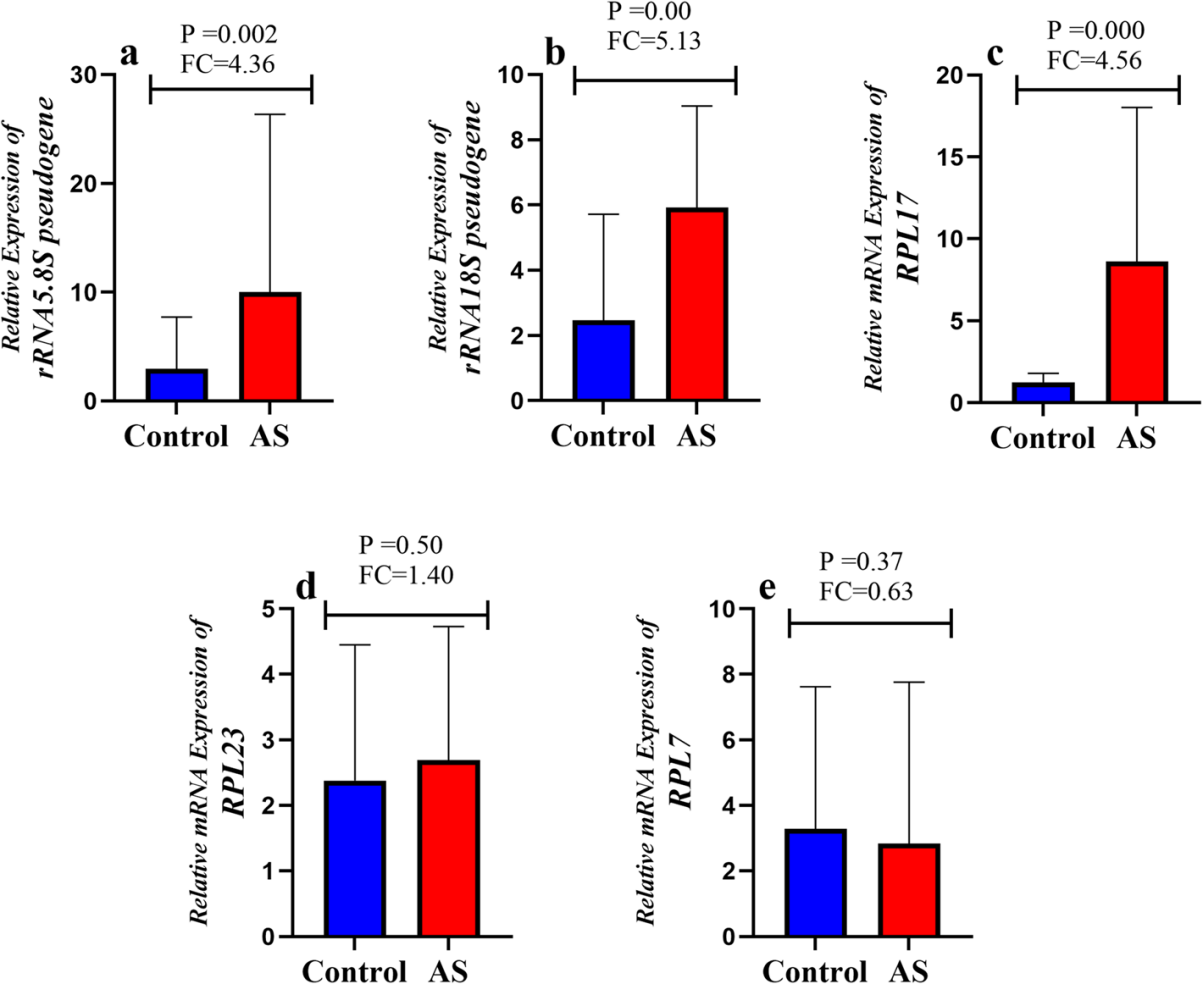
Bar graphs demonstrate the relative RNA expression (mean with SD) of (a) rRNA5.8S pseudogene; (b) rRNA18S pseudogene; (c) RPL17; (d) RPL23; and (e) RPL7, In 20 AS patients vs 20 healthy controls

Using RT-PCR assays for the certain genes, we confirmed that the expression level of *rRNA5.8S* pseudogene (fold change = 4.36, *p-value* = 0.002; Fig. 3a), *rRNA18S* pseudogene (fold change = 5.13, *p-value* = 0.000; Fig. 3b) and *RPL17* (fold change = 4.56, *p-value* = 0.000; Fig. 3c) in AS patients were significantly higher than controls, and will be discussed in the next section. mRNA expression levels of *RPL7*, and *RPL23* were not different between two groups (Fig. 3d and e).

## Discussion

The eukaryote ribosome is macromolecular complex, consisted of 2 subunits, the small 40S ribosomal subunit contains one 18S rRNA and 33 ribosomal proteins (RPS), whereas the large 60S subunit including the 28S, 5.8S, and 5S rRNAs together with 47 ribosomal proteins (RPL) [33]. A variety of extra-ribosomal functions for different ribosome’s components were recently confirmed, including the regulation of immune signaling, cellular development, cell cycle progression, and axial skeleton formation. Therefore, dysregulated expression of different ribosomal components elicits a wide spectrum of phenotypes, from developmental defects to diseases [12, 34]. Indeed, an entire class of diseases originates from defects in ribosome biogenesis called ribosomopathies, like Shwachman Diamond syndrome, Diamond-Blackfan anemia, and Dyskeratosis congenita. Despite the unique nature of their biochemical process, the clinical manifestations are extremely variable [35, 36].

Moreover, cancer cells are constantly associated with an increase in protein synthesis and dysregulation of ribosome biogenesis [13, 37]. RP altered expression pattern has been found in various human cancers, including the pancreas, breast, brain, bladder, and many others which could serve as prognostic or diagnostic markers [12, 16, 38, 39]. The increased expression of ribosomal protein mRNAs has been reported in human colon cancer and hepatocellular carcinoma [40]. Noteworthy, some ribosomal proteins like S13 and L23, provoke multidrug resistance in gastric cancer cells [41]. Furthermore, previous studies have linked increased rRNA expression with cancer development in cervical and prostate cancer [42, 43]. Overexpression of the pre-45S rRNA encourages G1/S cell-cycle transition in colorectal cancer and it is related to poor prognosis [44].

Ribosome-related networks have also been identified to be associated with different autoimmune rheumatic disorders. In a study by Luan et al. it was shown that ribosome-related pathway is associated with susceptibility to systemic lupus erythematosus (SLE), rheumatoid arthritis (RA), and multiple sclerosis (MS), suggesting that these diseases are associated to ribosomal genes [45]. Many of the auto-antibodies and auto-reactive T cells in different autoimmune rheumatic diseases recognized ribosomal proteins as an auto-antigens. It was reported that despite the similar frequency of anti-ribosomal antibodies in RA and SLE, they have different specific targets. Po, L7, L5, Sb, S19, S13, and L2 proteins in SLE, and L35a and L37a in RA identified by immunoblotting after two-dimensional gel electrophoresis with anti-ribosome antibodies [46, 47]. Some studies recognized differential ribosomal gene expression profiles in AS patients. In a recent study, Gracey et al. performed sex-dependent functional network analysis in AS patients and healthy controls and found translation/ribosome–related pathways dysregulated especially in male AS patients [48]. In a meta-analysis of differentially expressed genes and associated biological pathways in AS, was found that S11, L27, and L40 mitochondrial ribosomal proteins are among the most significant downregulated genes in AS patients [49].

In this study, *RPL17, RPL23A,* and *RPL7* were the three highly differentially expressed genes in our GSE analysis of the microarray dataset. Among the genes, *RPL17* was also significantly up-regulated in PBMCs from AS patients in the laboratory. Also in a previous bioinformatics analysis on GSE 25101 by Zhao et al. to predict the related genes to AS, RPL17 was among the ribosomal protein which significantly enriched in the selected module of the up-regulated network [50]. *RPL17* gene encodes a protein in humans called 60S ribosomal protein L17. In 2012, an extra-ribosomal function in vascular smooth muscle cells (VSMC) growth was reported for RPL17. Its expression was oppositely correlated with VSMC growth by increased cells in G0/G1 and decreased cells in the S phase. This study indicates that RPL17 could play a part in angiogenesis suppression, however, the exact mechanism remains unclear [51]. It was also involved in Diamond-Blackfan anemia and was reported to have higher expression in tumors across one cancer cohort [52].

RPL23A is one of the components of the large 60S ribosomal subunit and has been expressed in various organs at a high level. RPL23A can stimulate arthritogenic T cells differentiation to effector T lymphocytes and the production of IL-2 and inflammatory cytokines from mice. RPL23A has been expressed in fibroblast-like synoviocytes (FLS) in healthy and arthritis synovial tissues and is recognized by autoantibodies from patients with auto-inflammatory RA disease. The proportion of anti-RPL23A antibody-positive individuals is significantly higher in the RA patients group compared to healthy controls. Besides, a subset of T cells from RA patients produce interferon-γ upon RPL23A exposure [53, 54]. *RPL7* is a Protein Coding gene which is associated with systemic autoimmune diseases, such as SLE and other connective tissue diseases [55]. In contrast with our bioinformatics analysis, we did not find significant differences in *RPL23A* and *RPL7* expression in PBMCs from AS patients in the experimental section, and further investigations are needed for determining their role in the pathogenesis of AS.

In the current study, we also discovered overexpression of *rRNA5.8S* and *rRNA18S* pseudogenes in the bioinformatics section and also in the lab in PBMCs of AS patients. Due to the high similarity of pseudogenes to their genes, there is the possibility of binding of the pseudogenes designed primers to the *5.8S rRNA* and *18S rRNA* genes. Both of the ribosomal RNA is part of the 45S precursor which is transcribed by RNA polymerase I and also contains 28S rRNA [56]. In the eukaryotic ribosome, 5.8S rRNA is a non-coding component of the large subunit (60S) and previous studies showed its function in cell growth and protein synthesis [57]. Additionally, the covalent binding of the 5.8S rRNA to p53 was previously revealed and it was suggested that p53 and the RNA could be two integral parts of a single functional entity [58]. 18S rRNA is the structural RNA in small subunit (40S) of the eukaryotic cytoplasmic ribosomes [59]. Bowen-Conradi syndrome is a disease caused by a single mutation in EMG1. The gene is required for the assembly of 40S ribosomal subunit and is involved in the modification of the 18S rRNA [60]. In that patients, skeletal dysmorphology is observed and serious prenatal and postnatal growth defect usually leads to death by 1 year of age [61, 62].

Pseudogenes are non-coding alleles of normal genes that have long been considered as nonfunctional inactive elements, however, recent evidence demonstrated that many of them are transcribed into RNA, functionally active, and can regulate gene expression as long noncoding RNAs (lncRNAs) [63]. Pseudogenes have tissue-specific expression pattern and altered expression of them are involved in various diseases and physiological condition [64]. They play a crucial role in the immune response and are associated with signaling pathways involved in inflammation. The transcription of diverse pseudogenes is induced by pro-inflammatory cytokines such as tumor necrosis factor- α (TNF-α) [65]. There are not any previous reports, to our knowledge, investigating the expression of ribosomal RNA in AS, while some evidence indicates the ribosomal pseudogenes function in immune responses. In this line, it is demonstrated that 5S ribosomal RNA pseudogene 141 plays an essential role in antiviral innate immunity and induction of cytokine response by binding to retinoic acid-inducible gene-I (RIG-1) receptors [66].

The relation between epigenetic changes, such as DNA methylation and autoimmunity has been well established in the literature [67]. Moreover, global DNA hypo-methylation was reported in rheumatic diseases such as RA and lupus-like disease compared to the healthy group [68]. Furthermore, in another study, the association between AS and DNA methyltransferase 3A (DNMT3A), DNMT3B and DNMT3L, reinforce the notion that methylation variation is involved in AS pathogenesis [69]. In this regard, our finding of the dysregulation of ribosomal genes might be a confirmation for methylation changes that influence the expression of affected genes and consequently protein translation; although, further studies are needed in order to assess this hypothesis.

Strong evidence disclosed the integral role of ribosomal RNA and protein in various physiological and pathological processes associated with AS like innate immune response that regulate inflammatory cytokine induction (NF-kB, interferon-g (IFN-g)), inflammatory signaling, chondrocyte growth, and skeletal development [34, 70–72]. There are also observations regarding the role of the ribosomal components in other autoimmune diseases like MS, RA, and SLE and also diseases with high incidence in AS patients like cancers and atherosclerosis [12, 45, 51, 73, 74]. However little is known about the link between ribosome biogenesis with AS and further studies will be required to determine the relevance of ribosomal genes in the disease.

This study focuses on selected ribosomal genes of the microarray and RNA-seq datasets and evaluated their expression in community samples of Iranian AS patients and healthy controls. However, some limitations should be noted. First, the datasets were different in terms of therapeutic criteria, whereas medical treatments might affect the RNA expression [75]. Second, ankylosing spondylitis mainly has comorbidity with other diseases like bowel inflammation, uveitis, psoriasis, and heart disease [76]. These diseases might also affect the biological pathways and gene expression. In this study, unfortunately, due to a lack of information, the possible role of these comorbid diseases in gene expression has not been considered. Third, the number of samples in the experimental section was limited and the results of the study may be affected by the experimental conditions. Therefore, it is suggested to evaluate the *RPL23* and *RPL7* genes expression once again with more samples and by different experimental methods. These two genes showed significant dysregulation in patients compared to controls in the microarray dataset, while they did not confirm in laboratory analysis.

## Conclusion

In summary, our results identify the altered expression of some ribosomal components in bioinformatics and experimental analysis in AS patients. However, it is not clear whether the alternations in gene expression are the cause or effect of the disease. Knowing the significant contribution of ribosomal gene expression variation in diseases and biological processes associated with AS, no wonder that ribosome dysregulation could in part account for AS pathogenesis.

## Supplementary Information

**Additional file 1: Supplementary File 1**.pdf. Characteristics of subjects involved in the microarray dataset.

**Additional file 2: Supplementary File 2**.pdf. Detailed information of patient samples

## Data Availability

All data generated or analyzed during this study are available upon request.

## Abbreviations

AS: Ankylosing spondylitis
ASAS: Assessment of Spondyloarthritis International Society
BASDAI: Bath Ankylosing Spondylitis Disease Activity Index
BASFI: Bath Ankylosing Spondylitis Functional Index
BASMI: Bath Ankylosing Spondylitis Metrology Index
DNMT: DNA methyltransferase
EDTA: Ethylendiamine tetraacetic acid
FLS: Fibroblast-like synoviocytes
GEO: Gene Expression Omnibus
GSEA: Gene Set Enrichment Analysis
HLA: Human leukocyte antigen
HPRT1: Hypoxanthine phosphoribosyl transferase 1
IFN-g: Interferon gamma
lncRNAs: Long noncoding RNAs
MS: Multiple sclerosis
MSigDB: Molecular Signatures Database
NES: Normalized enrichment score
PBMCs: Peripheral blood mononuclear cells
PCR: Polymerase chain reaction
RA: Rheumatoid arthritis
RIG-1: Retinoic acid-inducible gene-I
RNP: Ribonucleoprotein
RPL: Ribosomal proteins of large subunit
RPS: Ribosomal proteins of small subunit
RPs: Ribosomal proteins
RRC: Rheumatology research center
rRNAs: Ribosomal RNAs
SLE: Systemic lupus erythematosus
TNF-α: Tumor necrosis factor alpha
TPM: Transcript per million
VSMC: Vascular smooth muscle cells
