# Supplementary material for: Dysregulation of ribosome-related genes in ankylosing spondylitis: a systems biology approach and experimental method
Source: BMC Musculoskelet Disord. 2021 Sep 14;22:789. doi: 10.1186/s12891-021-04662-2 (PMC8442383; doi:10.1186/s12891-021-04662-2)
Supplement: Supplementary file 1 — Additional file 1: Supplementary File 1.pdf. Characteristics of subjects involved in the microarray dataset. [file 12891_2021_4662_MOESM1_ESM.pdf]

Supplementary File 1. Characteristics of patients involved in the microarray dataset.

Characteristics	AS Patients
Male:Female (%)	55.6:44.4
Age (years; mean±SD)	45.9±12.9
AS duration (years; mean±SD)	16.0±12.9
Familiar history [n(%)]	5 (27.8)
BASDAI (mean±SD)	5.9±1.3
BASFI (mean±SD)	6.0±2.3
BASMI (mean±SD)	4.6±2.9
mSASSS (mean±SD)	17.3±21.5

BASDAI - Bath Ankylosing Spondylitis Disease Activity Index; **BASFI** – Bath Ankylosing Spondylitis Functional Index; **BASMI** - Bath Ankylosing Spondylitis Metrology Index; **mSASSS -** modified Stoke Ankylosing Spondylitis Spinal Score.

No TNF, corticoid, or methotrexate-treated patients were included.
