# Supplementary material for: Dysregulation of ribosome-related genes in ankylosing spondylitis: a systems biology approach and experimental method
Source: BMC Musculoskelet Disord. 2021 Sep 14;22:789. doi: 10.1186/s12891-021-04662-2 (PMC8442383; doi:10.1186/s12891-021-04662-2)
Supplement: Supplementary file 2 — Additional file 2: Supplementary File 2.pdf. Detailed information of patient samples [file 12891_2021_4662_MOESM2_ESM.pdf]

Supplementary File 2. Characteristics of patients involved in the RNA-seq dataset.

**Patient Samples**	**Ancestry**	**Gender**	**Age**	**Drugs**
GSM3308475	Caucasian	Female	33	Celebrex, Prednisolone, Golimumab, sulfasalazine, Escitalopram
GSM3308483	Caucasian	Female	41	Golimumab, Esomeprazole
GSM3308485	Caucasian	Female	28	Etanercept, OCP, Methotrexate
GSM3308486	Caucasian	Female	47	Etanercept, Padol fexofedine
GSM3308488	Caucasian	Male	45	Methotrexate (Maybe), Infliximab
GSM3308489	Caucasian	Male	35	Infliximab
GSM3308515	Caucasian	Male	32	Adalimumab, Celebrex (Maybe)
GSM3308516	Caucasian	Male	48	Infliximab, Sulfasalazine, Ibuprofen, digesic, PPI , Anti-hypertensive, zolendronic acid
